# Modalities for the Assessment of Burn Wound Depth

**Published:** 2006-02-15

**Authors:** Lara Devgan, Satyanarayan Bhat, S. Aylward, Robert J. Spence

**Affiliations:** The Johns Hopkins Burn Center/Michael D. Hendrix Burn Research Center, Baltimore, MD

## Abstract

**Objective:** Burn wound depth is a significant determinant of patient treatment and morbidity. While superficial partial-thickness burns generally heal by re-epithelialization with minimal scarring, deeper wounds can form hypertrophic or contracted scars, often requiring surgical excision and grafting to prevent a suboptimal result. In addition, without timely intervention, more superficial burn wounds can convert to deeper wounds. As such, the rapid and accurate assessment of burn wound depth is a priority in treating burn-injured patients. The object of this article is to review current research on modalities useful in the assessment of burn wound depth with emphasis on the relative costs and benefits of each technique. **Methods:** PubMed and Cochrane computerized databases were used for data retrieval, using the search terms “burns,” “burn wounds,” “burn depth,” “burn depth measurement,” and “burn depth progression.” In addition, bibliographic references from prior reviews of burn depth were reviewed. All peer-reviewed, English-language articles relevant to the topic of burn depth measurement were reviewed, including those focusing on animal and human populations. Where appropriate, conclusions drawn from review articles and expert analyses were included. **Results:** Although bedside evaluation remains the most common modality of diagnosing the depth of burn wounds, recent technological advances have broadened the scope of depth assessment modalities available to clinicians. Other depth assessment techniques include biopsy and histology, and perfusion measurements techniques such as thermography, vital dyes, indocyanine green video angiography, and laser Doppler techniques. **Conclusion:** Of the depth assessment modalities currently used in clinical practice, LDI and ICG video angiography offer the best data-supported estimates of accuracy. Until the future of new modalities unfolds, a combination of clinical evaluation and another modality—thermography, biopsy, or, ideally, ICG video angiography or LDI—is advised to best assess the depth of acute burn wounds.

Burn wounds are stratified into 4 categories of increasing depth: epidermal, superficial partial-thickness, deep partial-thickness, and full-thickness.^1^ Epidermal and superficial partial-thickness burns may be treated nonoperatively and will generally heal without scarring. Deep dermal and full-thickness burns, on the other hand, require more aggressive intervention. These are more morbid wounds wrought with hypertrophic scars and contractures, and they necessitate early surgical excision and grafting to optimize the outcome.^2^

In clinical practice, the depth of the burn wound is ultimately defined by the time to healing and the development of scarring. If a partial-thickness burn heals before 2 weeks after the burn injury, scarring is unlikely. Healing between 2 and 3 weeks increases the likelihood of scarring, and after 3 weeks, scarring is very likely. For those burn wounds that are not excised and grafted, the clinical course defines the original depth of burn.

Strict categorization of burn depth is confounded by *burn wound conversion*, a process by which superficial burns progress into deeper wounds via mechanisms that are not fully understood.^3^ Jackson first alluded to the pathogenesis of burn wound conversion via an elegant description of 3 concentric zones of burn injury: an irrevocably damaged *zone of coagulation*, a threatened but still viable *zone of stasis*, and an edematous *zone of hyperemia*.^3^ Via a series of interrelated mechanisms involving compromised perfusion, oxidative damage, and cytokine-mediated response, tissue in the zone of stasis may denature, thereby increasing burn wound depth. Recent literature has suggested that timely intervention may retard the rate and extent of burn wound conversion.^4^ As such, rapid and reliable methods for assessing burn wound depth are of great importance.

## CLINICAL EVALUATION

Bedside clinical evaluation is the most widely employed and cost-effective method of assessing burn wound depth.^5^ This method is based on the subjective assessment of visual and tactile characteristics of a burn wound, namely wound appearance, capillary blanching and refill, capillary staining, and burn wound sensibility to light touch and pinprick.^6^,^7^ Because the aforementioned characteristics are readily observed without preparation or advanced instrumentation, clinical depth assessment can be performed immediately, easily, and without cost.

Clinical evaluation has also been described using digital photographic images in lieu of bedside examination. Judgment based on digital photographs has been reported to have good agreement with bedside assessment.^8^ With transmission of noncompressed bitmap images averaging 1500 Kb or compressed images averaging 30 Kb, burn depth estimation is 90% accurate as compared to that with clinical diagnoses.^9^ Although this is a useful adjunct when remote consultation is required or desired, even high-resolution digital images can limit 3-dimensionality, and all digital images exist outside of the context of tactile examination.

Digital imaging aside, clinical evaluation as a mechanism of assessing burn wound depth has 2 significant limitations. First, the accuracy of bedside depth assessment is widely considered to be suboptimal.^10^,^11^ Although clinical judgment can diagnose very deep and very shallow burns with strong validity, it is markedly less accurate for burns of intermediate depth.^5^ Overall estimates report that clinical depth assessment is accurate in about two thirds of cases,^12^ with the most frequent cause of error attributed to depth overestimation.^13^

The second limitation of clinical assessment centers on validity of diagnosis. Reliability is questionable in bedside evaluation of burn depth, and considerable variation exists between assessments by different clinicians.^14^ Not only is the baseline level of experience with burn assessment variable, the extent of tissue damage may not be immediately visually apparent as well.^1^

## BIOPSY AND HISTOLOGY

Punch biopsy of burned tissue with subsequent histological assessment has been regarded by some as the criterion standard of depth diagnosis, serving as the basis for comparison of other diagnostic modalities.^5^,^10^ Biopsy reflects the patency of dermal vessels and the structural integrity of interstitial and cellular proteins, with occluded vessels and denatured proteins indicative of devitalized tissue.^7^ It has been argued that microvascular damage suggests that a burn is partial-thickness, whereas collagen denaturation suggests that it is full-thickness.^15^

Although biopsy with histological analysis is a well-studied and widely accepted method for depth assessment, it is not without disadvantages. Despite biopsy's place as the criterion standard of depth diagnosis, it is not inherently 100% accurate. Sampling error, which occurs when a nonrepresentative portion of the wound is biopsied, and tissue shrinkage, which occurs when a specimen is histologically mounted, are of concern.^7^ Structural tissue damage may not necessarily correlate with functional loss in the acute time frame, which implies that early biopsies are not wholly accurate, particularly in light of the progressive nature of burn wounds and burn wound conversion.^16^

Beyond the inaccuracies fundamental to biopsy, the most notable limitation is the invasive nature of the procedure. Because burns can have marked variation in vessel patency, multiple biopsies of different wound areas may be required, thereby causing added scarring and increasing the risk for infection.^7^ Employing biopsy as a depth assessment technique is also limited by the need for an experienced pathologist to interpret specimens. Moreover, biopsy interpretation itself is subjective from a histopathological point of view.^17^

## MEASUREMENT OF TISSUE PERFUSION

### Thermography

Thermography is based on the measurement of burn wound temperatures as an indicator of their depths. By exploiting the notion that deeper wounds are colder than more superficial ones because of less vascular perfusion near the wound surface, thermography is able to inversely correlate temperature with depth.^18^ Still et al report that the accuracy of thermography is as high as 90% based on 1 degree differences in temperature at various aspects of a wound.^6^

Although thermography benefits from relative technical ease and validity, it is limited by the confounding effects of ambient heat loss and sensitive timing. Evaporative loss of heat to the environment causes wounds to be interpreted as falsely deep, introducing systematic error to this technique. In addition, accuracy is compromised if wounds begin to granulate, so optimal results occur when thermography is done within 3 days of sustaining the burn injury.^19^

### Vital dyes

Fluorescent and nonfluorescent vital dyes are inexpensive but rarely used modalities of assessing burn wound depth. Fluorescein fluorescence involves intravenous injection of fluorescein dye, followed by illumination with 360 to 400 nm ultraviolet light over burned areas. Ultraviolet light aids in depth visualization, but because it incompletely penetrates soft tissue, fluorescein fluorescence can neither differentiate between superficial and deep partial-thickness burns nor detect viable tissue that is masked by overlying eschar.^2^ In addition to diagnostic limitations, fluorescein dosing is limited by renal clearance and capillary leak.^6^

Nonfluorescent vital dyes, specifically Evans blue, patent blue V, and bromophenol blue, have also been studied for use in burn depth assessment.^2^ Although vital dyes can identify surface necrosis, they are generally not able to distinguish between partial- and full-thickness burns.^6^ Given the limited amount of diagnostic information vital dyes provide, they are regarded as having low clinical utility.

### Indocyanine green video angiography

Indocyanine green (ICG) was initially investigated as a means of measuring tissue perfusion using still images produced by fluorescent angiography.^20^,^21^ Recent modification of this technique using videography allows the capturing of dynamic changes in tissue perfusion.^22^ Following an intravenous dose of ICG, laser fluorescence videography is used to create a video image of dye uptake and clearance as an indicator of tissue perfusion. Normal unburned skin is used as a control to normalize measurements. Given that impaired microcirculation is directly associated with tissue necrosis, data from ICG video angiography can determine dermal viability with high sensitivity.^22^ Indeed, ICG video angiography has also been reported as an accurate means of measuring regional blood flow in nonburn patients.^23^

In addition to its diagnostic utility, ICG video angiography has the advantage of being able to correlate structure (ie, coagulated dermal vessels) with function (ie, impaired local perfusion).^23^ It also has the benefit of being a benign, well-studied substance that is hepatically cleared.^21^

One significant limitation of ICG video angiography is that topical ointments, dressings, and blood interfere with the measurements it produces. According to Haslik et al, these superficial substances can decrease absorption measurements by 63% ± 36%, thereby dramatically overestimating the depth of burn wounds.^24^ Current practice standards suggest complete removal of all topical substances from a wound at least 10 minutes prior to ICG video angiography to avoid this loss of accuracy.^25^ As with other advanced techniques for burn depth assessment, widespread use of ICG video angiography is limited by the somewhat expensive and sophisticated infrastructure it requires.

### Laser Doppler techniques

Laser Doppler flowmetry and laser Doppler perfusion monitoring (LDPM) are the oldest Doppler-based techniques used for burn depth estimation. Doppler flowmetry is based on the Doppler principle, which states that when mono-frequency light waves are reflected off moving objects, they undergo a change in frequency. By analogy, laser light that is directed at moving blood cells in sampled tissue will exhibit a frequency change that is proportional to the amount of perfusion in the tissue.^25^ Using a fiber-optic probe in direct contact with the burn wound, this technique assesses microcirculation 1 mm below the point of probe-tissue contact.^26^ The accuracy of flowmetry in burn depth diagnosis ranges from 90% to 97%, as compared to 66% with clinical evaluation alone.^10^,^27^,^28^ In addition, the positive predictive value of laser Doppler flowmetry is as high as 98.4%.^29^

Despite this high accuracy, technical limitations render flowmetry a suboptimal technique. Because flowmetry requires that the probe be in direct contact with the burn wound, it increases the risk of wound infection and inflicts trauma to already vulnerable tissue.^29^ In addition, because it measures perfusion in one spot at a time, assessing a large burn wound can be a lengthy and unwieldy process.^30^

Laser Doppler imaging (LDI) and laser Doppler perfusion imaging (LDPI) overcome the limitations of flowmetry by exploiting a noncontact scanning technique that allows measurement of the entire burn wound surface. After scanning a burned area, LDI devices generate a color-coded perfusion map that corresponds to varying burn depths. LDI is a highly valid measure of burn wound depth, and its accuracy has been reported at up to 99% if infected wounds are excluded.^31^ Indeed, the most recent studies addressing the critical interface between superficial and deep partial-thickness burns suggests that LDI can reliably predict the level that distinguishes between burns that will or will not heal by re-epithelialization by 3 weeks.^10^ Adjunct measures such as sequential scans, heat-provocation scans, and digital imaging can increase test validity.^11^ In addition, by avoiding direct tissue contact, LDI and LDPI minimize patient discomfort, infection risk, and tissue microtrauma.^11^

The major limitation of LDI and LDPI is that their accuracy is contingent on optics. That is, because LDI and LDPI are optics-based techniques, heterogeneity and curvature of tissues, topical substances, ambient light, and wound infection can adversely affect depth measurements.^32^ Nevertheless, despite their significant expense, LDI and LDPI are the latest, most accurate, and most advanced modalities for diagnosing burn wound depth.

## NOVEL TECHNIQUES AND FUTURE DIRECTIONS

With the advent of ICG video angiography and noncontact laser Doppler techniques, the assessment of burn wound depth is becoming an ever more accurate and sophisticated endeavor. Indeed, several novel techniques have been described as possible future directions in the area of burn depth assessment. Although not yet clinically demonstrated, optical measurement, nuclear imaging, and noncontact ultrasound hold promise for the future of estimating the depth of burn wounds.

### Optical measurement

By denaturing cellular proteins, burns alter the tissue structure and optical properties. Several novel techniques have been developed to quantify burn-induced optical variations as indicators of wound depth. Although none has been proven valid in a clinical setting, each technique is currently being studied with that end in mind.

Reflection-optical multispectral imaging performs a spectral analysis of reflected light from burn wounds, with the concept that necrotic tissue, scarring, and dermal vessel oxygen saturation alter absorption.^32^ Reflection-optical multispectral imaging industrial prototypes are currently being used in various burn centers in an effort to determine clinical validity.

Optical coherence tomography uses polarity measurements of birefringence amplitude, orientation, and diattenuation to assess tissue structure and function.^33^ Polarization-sensitive optical coherence tomography measures the extent to which reflected light from burns has changed polarity. Reduction in collagen birefringence is thought to be related to burn depth. While polarization-sensitive optical coherence tomography has been studied in animals, it has not been demonstrated in humans.^34^

Fiber-optic confocal imaging involves noninvasive microscopy of subsurface living tissue. Illumination of tissue with blue light frequencies causes autofluorescence that is directly proportional to the burn depth.^35^ It is thought that the mechanism of autofluorescence is related to the presence of denatured collagen or other cellular proteins. Data for fiber-optic confocal imaging is based on animal models, and clinical trials are yet to be performed.

Orthogonal polarization spectral imaging illuminates the tissue with polarized light within the hemoglobin spectrum. It has been used for the noninvasive assessment of skin microcirculation through the surface of the human burn wound.^36^ Using this technique, 2 distinct microcirculatory patterns were seen in burned skin: superficial burns had small visible dermal capillaries studded throughout the field of view, while deep burns showed large thrombosed vessels coursing in a criss-crossed fashion. This disparity reflects the marked difference between the mean optical densities for superficial burns and deep burns. Orthogonal polarization spectral imaging is limited as a technique in that it requires direct tissue contact and covers only a small area in one reading.

While still in their infancy, these optical techniques offer the promise of noninvasive, noncontact, rapid assessment of burn wounds. As animal and human studies progress, these modalities may become the next innovation in burn depth diagnostics.

### Nuclear imaging

Radio-labeled tracers have been used to map burn depth in the context of animal models. Sayman et al described the use of a ^99m^Tc methoxyisonitril (MIBI) tracer to delineate areas of muscular burns in a rat model.^37^ As expected, decreased perfusion in burned tissue manifested itself as decreased presence of the radiotracer. Although highly sensitive for burn depth in this animal model, the use of radioactive tracers may add an unnecessary potential morbidity to already compromised patients. As nuclear imaging becomes more affordable and widespread, this modality may become more important as a rapid, noninvasive depth diagnosis technique.

### Noncontact and high-frequency ultrasound

While traditional ultrasound requires dermal contact, noncontact ultrasound functions via a probe that is held 1 in from skin.^38^ Using this novel device, the operator is able to reliably distinguish epidermis, dermis, and dermal-fat interface in burned skin. With the assumption that the visualized dermal-fat interface corresponds to the depth of a full-thickness dermal burn, the operator is able to estimate the depth of tissue injury with reasonable accuracy. Although not yet proven in humans, noncontact ultrasound has been demonstrated to be a rapid, accurate, noninvasive diagnostic tool in animal models.

High-frequency ultrasonography has also been suggested as a means of burn depth assessment. This modality uses a contact probe with frequencies in the range of 20 to 200 Hz to assess dermal and subdermal anatomic features. Although it requires direct skin contact, high-frequency ultrasound is able to identify dermal adnexal structures with improved resolution, thereby offering a potentially more accurate visualization of deep dermal microcirculation.^39^ However, the extent to which high-frequency ultrasound can differentiate between deep dermal vasculature and edema or inflammation is not clear.^39^ It has been suggested that the combination of high-frequency ultrasound with Doppler flow imaging would be a more accurate method of color flow microcirculation mapping.^40^ Although high-frequency ultrasound represents a theoretical improvement upon noncontact ultrasound and standard-frequency ultrasound techniques, it is limited not only in its requirement for tissue contact but also in its as yet unproven clinical ability.

## CONCLUSION

Assessment of burn wound depth remains an important clinical goal in the management of the acutely burned patient. Not only does depth dictate patient prognosis, it indicates the most appropriate clinical intervention for a given wound as well. As such, understanding the relative efficacies of various modalities for evaluating burn depth continues to be a priority.

Although bedside clinical evaluation remains the most widespread and cost-effective method for depth diagnosis, it is accurate only about two thirds of the time and is limited by poor interrater reliability. Thermography, though less frequently employed, is about 90% accurate. Its widespread acceptance is limited by evaporative heat loss confounding the reliability of its results. Punch biopsy has been considered the criterion standard of depth diagnosis; however, errors in sampling and histopathological interpretation imply that biopsy, too, has its shortcomings.

Newer developments in burn depth assessment focus on measurements of tissue perfusion. While fluorescent and nonfluorescent vital dyes have fallen out of favor due to their low sensitivities, ICG video angiography and LDI have fully supplanted them. Of depth assessment modalities currently used in clinical practice, LDI and ICG video angiography offer the best data-supported estimates of accuracy. ICG video angiography is unique in that it offers a dynamic portrait of vessel patency that fluctuates in real time. LDI provides a static rather than dynamic perfusion map, but it retains other advantages over ICG video angiography. It is not only less invasive and faster but also more accurate, with validity as high as 99%. Most important, it seems to be the best technique for assessing the depth of burn at the critical and most difficult to diagnosis level, between superficial and deep partial-thickness burns.

The future of depth assessment in burn wounds hinges on cost-control and further study of LDI and ICG video angiography, as well as on the development of novel techniques not yet proven in humans. Optical measurements and noncontact ultrasound offer the promise of rapid and noninvasive diagnostic studies with minimal patient morbidity. Nuclear imaging, on the other hand, sacrifices a modicum of morbidity but allows for the possibility of increased diagnostic precision. As future animal models better establish the efficacy of these techniques, clinical trials in human patient populations will be needed for further hypothesis confirmation.

Until the future of new modalities unfolds, a combination of clinical evaluation and another modality—thermography, biopsy, or ideally, ICG video angiography or LDI—is advised to best assess the depth of acute burn wounds.
